# Improving antimicrobial stewardship with penicillin allergy testing: a review of current practices and unmet needs

**DOI:** 10.1093/jacamr/dlac116

**Published:** 2022-11-19

**Authors:** Claude Mabilat, Marie-Françoise Gros, Alex Van Belkum, Jason A Trubiano, Kimberly G Blumenthal, Antonino Romano, Tristan T Timbrook

**Affiliations:** bioMérieux, Medical Affairs, 100 Rue Louis Pasteur, F-69280 Marcy l'Etoile, France; bioMérieux, Medical Affairs, 100 Rue Louis Pasteur, F-69280 Marcy l'Etoile, France; bioMérieux, Open Innovation and Partnerships, 3 Route du Port Michaud, 38390 La Balme Les Grottes, France; Department of Infectious Diseases, Centre for Antibiotic Allergy and Research, Austin Health, 145 Studley Road, Heidelberg, Victoria, 3084Australia; Division of Rheumatology, Allergy, and Immunology, Department of Medicine, Massachusetts General Hospital, Boston, MA, USA; Harvard Medical School, Boston, MA, USA; Oasi Research Institute-IRCCS, Allergy Unit, Troina, Italy; bioMérieux, BioFire Diagnostics, Global Medical Affairs, 515 Colorow Drive, Salt Lake City, UT 84108, USA

## Abstract

Penicillin allergy, the most frequently reported drug allergy, has been associated with suboptimal antibiotic therapy, increased antimicrobial resistance, increased rates of *Clostridioides difficile* colonization and infection, as well as extended hospital length of stay and increased cost. Although up to 10% of all patients may report penicillin allergy, most penicillin allergies are not confirmed. As such, most patients with a penicillin allergy can still safely use penicillin and related drugs following a more precise assessment. Herein, we review the current practices and unmet needs in penicillin allergy testing.

The diagnostic algorithm is mostly based on a clinical history assessment followed by *in vivo* testing, i.e. skin test and/or drug challenge. As these tests are labour and resource intensive, there is increased interest in point-of-care penicillin allergy de-labelling solutions incorporated into Antimicrobial Stewardship Programmes including digital assessment tools. These can be locally parameterized on the basis of characteristics of target populations, incidence of specific allergies and local antibiotic usage to perform clinical risk stratification. Safely ruling out any residual risk remains essential and *in vivo* drug challenge and/or skin testing should be systematically encouraged. Gradual understanding and convergence of the risk stratification of the clinical presentation of penicillin allergy is enabling a wider implementation of this essential aspect of antimicrobial stewardship through digitalized decision tools and *in vivo* testing. More research is needed to deliver point of care *in vitro* diagnostic tools to democratize this de-labelling practice, which would be highly beneficial to patient care. This progress, together with better education of patients and clinicians about the availability, efficacy and safety of penicillin allergy testing, will increase the dissemination of penicillin allergy assessment as an important component of Antimicrobial Stewardship Programmes.

## Introduction

**Figure 1.**
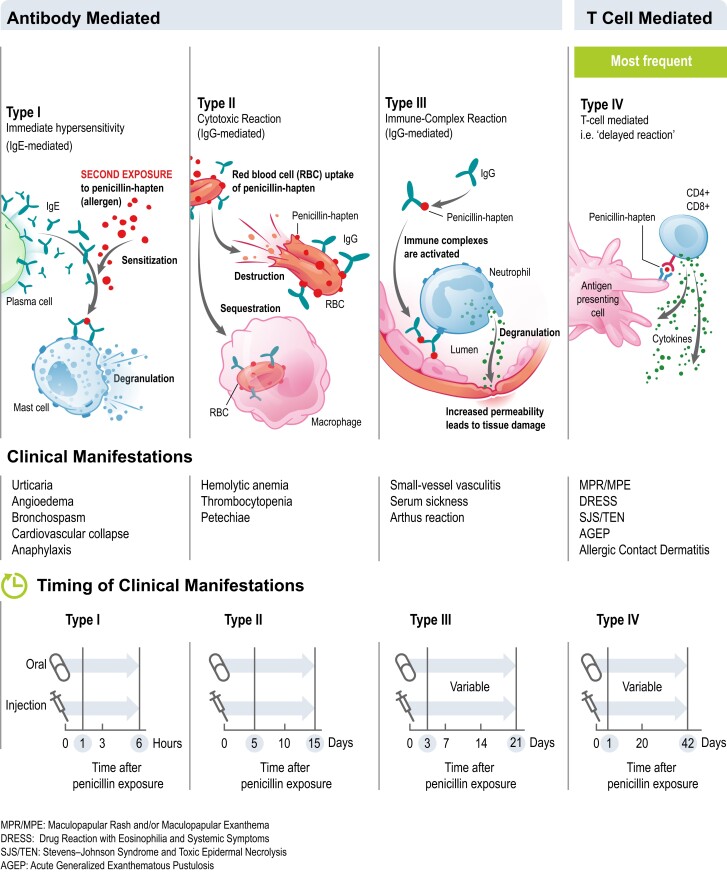
Mechanisms of hypersensitivity reactions to penicillin and clinical manifestations (adapted from Castells 2019).

Allergy is due to disordered activation of the immune system translating into hypersensitive responses to substances in direct contact with the human body. Allergies are triggered by specific substances originating from the environment (pollen, insect venoms, latex, etc.), foods (peanuts, shellfish, etc.) or drugs. Four types of hypersensitivity reaction (Types I–IV) were described in the Gell and Coombs classification, based on the underlying immune mechanisms (Figure 1).^1^

**Figure 2.**
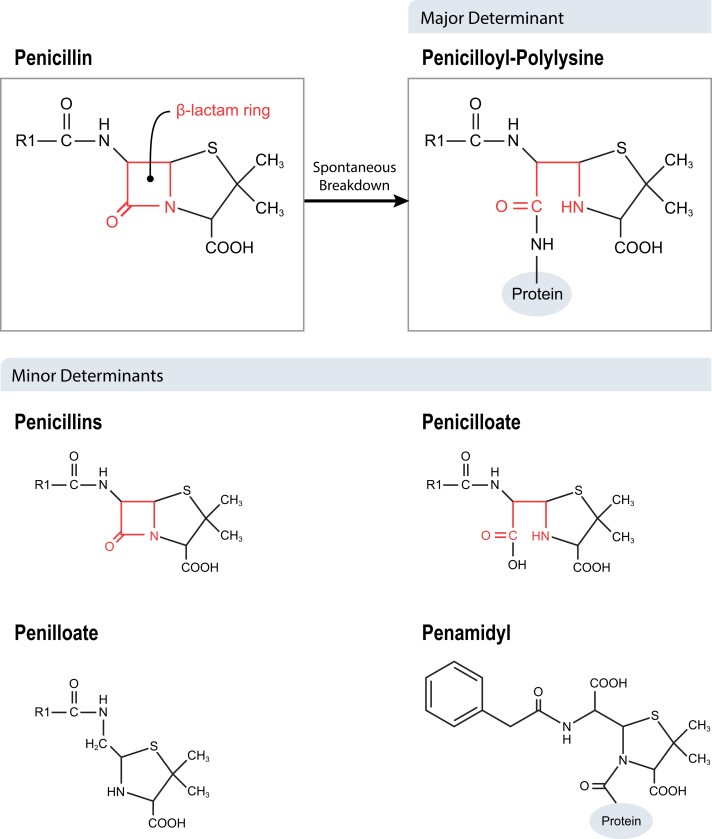
Penicillin allergenic derivatives (adapted from Matas 2018).

Penicillins are small molecules that bind to various human and microbial proteins and create hapten–carrier complexes. When metabolized, their β-lactam ring is a target for nucleophilic attack by free amino groups of proteins, leading to ring opening and covalent amide bonding of the penicilloyl group. The penicilloyl configuration, where the hapten determinant is covalently linked to amino groups of lysine residues of proteins, constitutes more than 90% of the reaction products between proteins and penicillins (Figure 2). The entire β-lactam family of antibiotics can cause allergy, in particular benzylpenicillin (penicillin G), oxacillin, semi-synthetic penicillins in combination with β-lactamase-inhibitors (e.g. amoxicillin/clavulanic acid, ampicillin/sulbactam and piperacillin-/tazobactam), as well as cephalosporins, monobactams and carbapenems.

**Figure 3.**
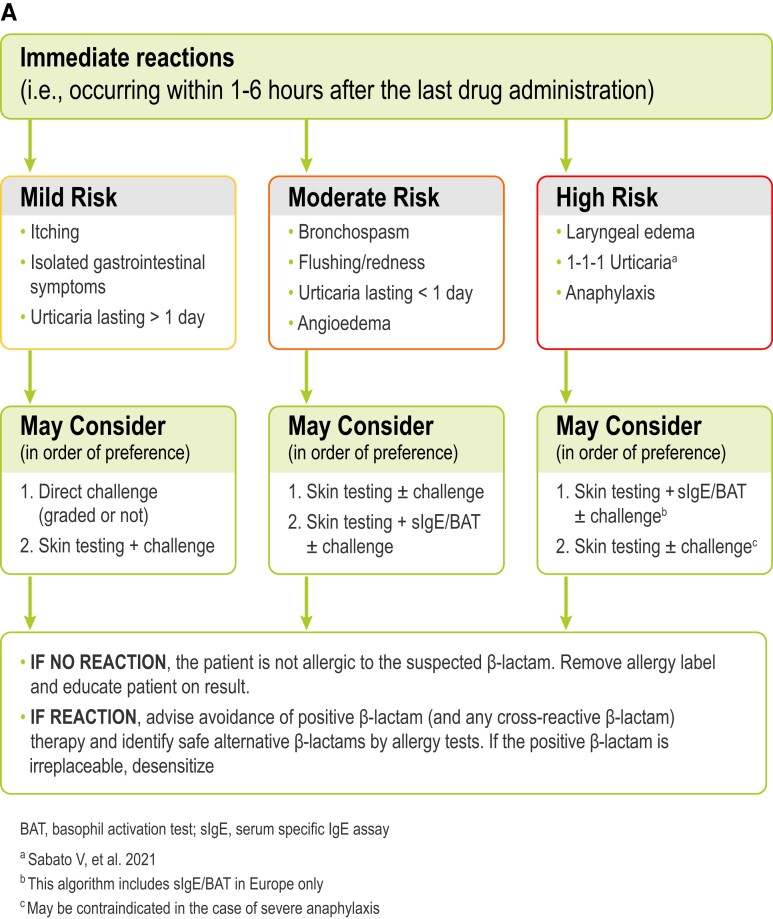

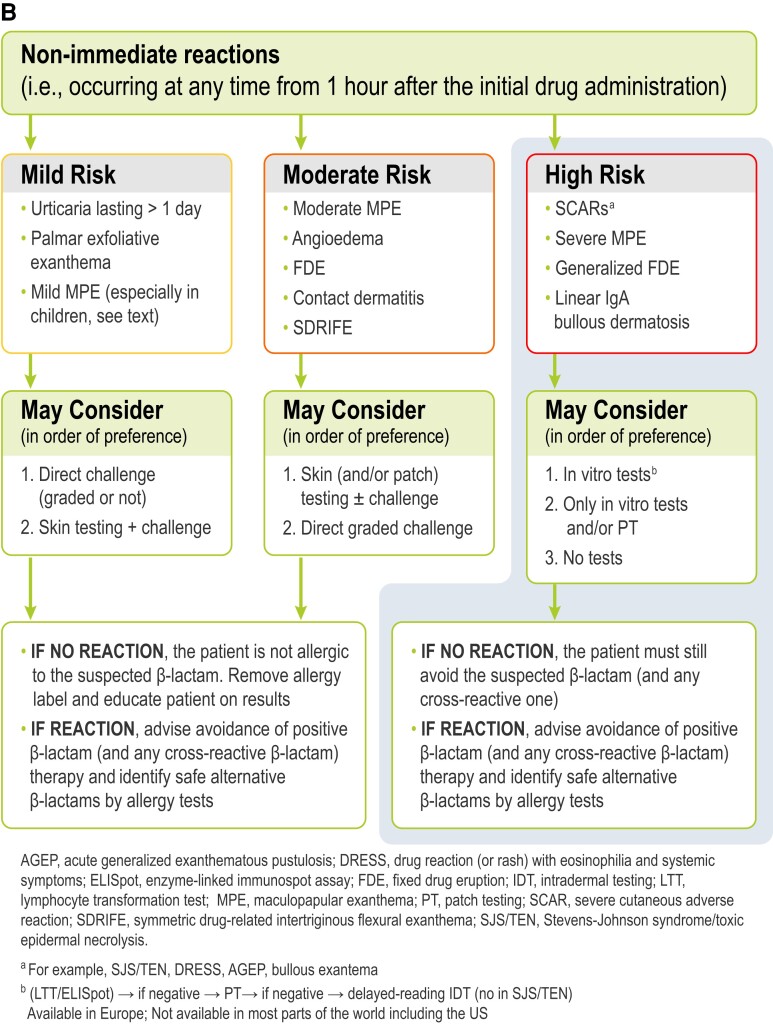
An example of a European penicillin allergy testing algorithm based on the patient risk stratification for (A) immediate and (B) non-immediate reactions.

The risk of cross-reactivity between penicillins and cephalosporins varies with the degree of similarity between R1 side chains, ranging from 2.1% for cephalosporins with low similarity scores to 16.4% for aminocephalosporins, which share very similar or identical side chains with aminopenicillins.^2^ In meta-analysis focused on penicillin and cefazolin only, dual allergy frequency was 0.7%.^3^ However, such frequency was lower for participants with unconfirmed (0.6%) versus confirmed penicillin allergy (3.0%). There is evidence that even in people with severe immediate phenotype (e.g. anaphylaxis), non-cross-reactive β-lactam antibiotics can still be used.^4,5^ In patients with a history of severe delayed phenotypes, such as severe cutaneous adverse reactions (SCARs: i.e., Stevens–Johnson syndrome-toxic epidermal necrolysis, drug reaction with eosinophilia and systemic symptoms syndrome, acute generalized exanthematous pustulosis), interstitial nephritis etc., the risk of cross-reactivity is less defined, with potentially broader cross-reactivity noted and, therefore, alternative β-lactam antibiotic use requires a risk/benefit assessment by an allergist. Figure 3 shows an example of risk stratification in β-lactam allergy and management. Subjects who suffered severe reactions, or who have a high probability of experiencing a reaction more severe (e.g. anaphylaxis) than the index reaction (e.g. urticaria) in case of re-exposure to the culprit β-lactam, are classified as being at high risk. Subjects who experienced mild/moderate reactions, or who have a low probability of experiencing a reaction more severe than the index reaction in case of re-exposure to the culprit drug, are classified as low risk. Moderate-risk individuals are those with an intermediate risk condition.

Herein, we address the clinical manifestations of the penicillin class antibiotic allergy, the de-labelling of incorrectly attributed penicillin allergies and the tools available to do penicillin allergy assessments. We will also define the medical needs, indicating the areas where further development of diagnostics that are rapid and *in vitro* could aid appropriate in antibiotic prescribing.^6,7^ Additionally, we discuss how the patient and clinician perspectives are important for facilitating the integration of these advances to fuel an elevated antimicrobial stewardship (AMS) practice.

## An over-labelled allergy: true versus reported

Symptoms of penicillin allergy range from mild rashes to severe and sometimes fatal reactions, including anaphylaxis (Figures 1 and 3). Reported penicillin allergy (i.e. true or not) is the most common drug allergy with a prevalence ranging from 6% to 25% across various regions and treatment populations.^8–11^ Studies have shown that less than 5%–10% of these penicillin allergy labelled individuals have a true allergy when assessed with a drug challenge test (DCT, also called the drug provocation test) after negative skin prick testing.^8,12,13^ This implies that more than 90% can still be treated safely with this class of antibiotics. However, this approach, which is considered the gold standard for penicillin allergy exclusion, although extremely effective, has been unable to fully address the substantial and growing need in the community, mainly because of a lack of accessible or timely allergy assessment services,^14^ and this may have contributed to reinforce the burden of spurious penicillin allergy labels. The low rate of true penicillin allergy found among individuals with this label can be explained by the fact that most diagnoses of penicillin allergy are made in childhood and relate to low-risk delayed cutaneous eruptions such as mild maculopapular exanthems (MPEs). Moreover, IgE-mediated hypersensitivity to β-lactams can wane over time.^5,12,15,16^ Some studies demonstrated that 20%–50% of patients with an IgE-mediated hypersensitivity to penicillins and/or cephalosporins may lose sensitivity and become skin-test negative within 1 year and more than 60% within 5 years.^17,18^ However, this loss of hypersensitivity over time is not always definitive, and while re-administration of the responsible penicillin can carry risks, this risk appears close to the baseline population risk.^19^ Indeed, there are studies^4,20–23^ in which subjects with a suspected hypersensitivity to β-lactams and negative allergy tests were re-evaluated within 2 to 4 weeks after negative challenges and/or therapeutic courses with the drugs involved and presented a re-sensitization with a frequency from 0.4%^22^ to 25%.^23^ In particular, in a recent study, 20 subjects with immediate reactions to cephalosporins and negative skin tests underwent re-evaluations after negative cephalosporin challenges: a conversion to cephalosporin skin-test positivity occurred in five of the six subjects who had had anaphylactic reactions and in none of the remaining 14 subjects with other (non-anaphylactic) reactions.^23^ The mean time interval between the most recent cephalosporin reaction and allergy examination in the 74 subjects with negative results to cephalosporin skin tests was 50.8 ± 77 months. In patients with low-risk penicillin allergies (Figure 3) who have been evaluated and de-labelled, re-sensitization or adverse events are rarely reported post-testing.^24,25^

## Why is penicillin allergy over-labelling a problem?

### Effects on patients

Observational studies have suggested poorer clinical outcomes among those with a penicillin allergy label, including increased in-hospital mortality and significantly prolonged length of hospital stay.^8,26^ Such patients frequently receive less effective second-line therapies or broader-spectrum initial antimicrobial agents due to their reported allergy that contributes to antimicrobial resistance.^10,27–29^ For example, a prospective cohort study showed that patients with a reported β-lactam who did not receive preferred β-lactam had a higher adjusted risk of adverse events (readmission, *Clostridioides difficile* infections, drug reaction, or acute kidney injury) than patients without a reported allergy.^30^

A retrospective study showed that patients with sepsis and a penicillin allergy label are less likely to receive the first dose of intravenous antibiotic within 1 hour of diagnosis and more likely to receive second-line antibiotics such as carbapenems and fluoroquinolones. They suffer a greater antibiotic burden, while incurring higher costs to the health service.^31^ There is still a gap in knowledge however, since it has not yet been established that de-labelling can help to reverse some of these complications.

### Effects on healthcare-associated infections

Penicillin allergy over-labelling has a strong impact on the development of hospital acquired infections. For instance, the prevalence of *Clostridioides difficile* infections increased by 23% and 26% in US and UK patients, respectively, who were admitted to hospital while being attributed with a penicillin allergy label compared to those without a label.^8,32^ This is most probably due to the subsequent use of β-lactam alternatives (e.g. fluoroquinolones) that had a detrimental impact on the gastro-intestinal microbiota. It is well established that alternative antibiotics such as fluoroquinolones have a higher association with *Clostridioides difficile* infections than β-lactams, although duration of exposure to these high-risk alternatives may be mitigated with antibiotic allergy evaluations.^33,34^ When patients with penicillin allergy labels acquire surgical site infections (SSIs) this is often due to inferior perioperative prophylactic antibiotic choice and timing. Among 8385 perioperative patients in the US, penicillin allergy labelling resulted in 50% increased odds of SSIs.^35^ The increased SSI risk was entirely explained by inappropriate choice of perioperative antibiotics.

### Effects on antimicrobial resistance

A UK study identified that a penicillin allergy label conferred a 69% increased incidence of methicillin-resistant *Staphylococcus aureus* where 55% of the increased risk was attributable to administration of alternative β-lactam antibiotics.^32^ A US study documented a 14% increased prevalence of methicillin-resistant *S. aureus* and a 30% increased prevalence of vancomycin-resistant *Enterococcus* in hospital inpatients with a presumed penicillin allergy.^8^

As most patients with a reported penicillin allergy could still safely tolerate β-lactams, promoting their use when patients are appropriately risk-stratified and have an obvious ‘low-risk’ penicillin allergy is increasingly regarded as part of AMS core strategies.^36,37^ Remarks to that extent are now integrated in the CDC Core Elements for Antimicrobial Stewardship in Hospitals (2019) as well as in the WHO Practical Tool Kit for Low and Middle Income Country Healthcare Facilities (2019).

## How is penicillin allergy diagnosed?

The evaluation of patients labelled as allergic to penicillin begins with an allergy risk assessment based on the clinical history that includes information on the chronology (i.e. immediate and non-immediate) and morphology (i.e. clinical signs and symptoms of the reaction: in particular, the type of cutaneous eruption. Figure 3), treatment of the reaction and relevant ingestions concurrent with, and since, the clinical reaction.^6,38^ There are no globally accepted and standardized tools although suggestions have been progressively adapted.^10,38–41^ A prediction tool entitled ‘PENicillin allergy, within last Five years, Anaphylaxis/angioedema, SCAR and Treatment required for allergy episode’ with the acronym PEN-FAST was developed in Australia and was locally and internationally (one US and European site) validated.^42,43^ PEN-FAST allows patients to be stratified into very low, low-, moderate- and high-risk groups on the basis of their respective allergy histories (risk of positive penicillin allergy test of <1%, 5%, 20% and 50%, respectively). For low-risk groups, PEN-FAST has been shown to provide a negative-predictive value (NPV) of 96%.^44^ PEN-FAST is currently being prospectively studied in an international specialist-led clinical trial.^45^

More than 80% of reported penicillin-induced reactions involve the skin.^8^ Penicillins were responsible for approximately 37% of 269 493 ‘rash/dermatitis’ cases and for 40% of 150 450 ‘hives/urticaria’ ones documented in electronic health records of a large US health system.^46^ There is no broad consensus on the risk stratification of subjects reporting cutaneous eruptions such as urticaria and MPE associated with penicillin therapy.^12,16^ Some authors classify subjects reporting rash as being at mild risk and those reporting urticaria as at moderate risk of penicillin allergy.^12^ Others consider urticaria and delayed MPE benign cutaneous reactions and classify subjects who report immediate isolated urticaria (≤6 hours after exposure), delayed isolated urticaria (>6 hours after exposure) or benign exanthema as being at low-to-medium risk of penicillin allergy.^16^ In any case, detailed information on penicillin-associated cutaneous eruptions is needed to facilitate research into this area of persistent uncertainty.

A recent European position paper recommended assessing the reaction history considering both the sign and symptoms of reactions and chronology.^5^ Drug reactions can be classified as immediate and non-immediate (often also called delayed). The former typically occur within 1 hour but may occur up to 6 hours after the last administered dose and are mostly associated with an IgE-mediated pathogenesis. Immediate reactions usually manifest as isolated symptoms, such as urticaria, angioedema, erythema/redness and bronchospasm/wheezing or as anaphylaxis. Non-immediate reactions may occur at any time from 1 hour after the initial drug administration, but commonly after many days of treatment, and are often associated with a T-cell-mediated pathogenic mechanism.^41^ MPE and delayed urticaria are the most common clinical presentations of non-immediate reactions. Because of the peculiarity of the morphological characteristics of most drug reactions, considering both their chronology and morphology can limit the number of possible overlaps in classifying them. In the case of an urticarial reaction occurring after more than 1 hour but within 6 hours of the first dose of a β-lactam, it is advisable to classify it as immediate. Another history characteristic of drug reaction is its duration. The recent European position paper classified MPEs associated with β-lactam therapy as severe, moderate or mild.^6^ The first consist of widespread rashes lasting more than 1 week and with systemic involvement (e.g. fever, eosinophilia); the second and the third are widespread rashes lasting more than 1 week and less than 1 week, respectively, and without systemic involvement.

The characteristics of urticarial reactions reported by 410 subjects during β-lactam therapeutic courses were correlated to the results of a systematic allergy workup, which included skin tests, serum specific IgE (sIgE) assays and challenges.^47^ Urticaria with an onset within 1 hour (OR: 17, 95% CI: 9–31, *P* < 0.001) after the first dose (OR: 11, 95% CI: 6–20, *P* < 0.001), and with a maximal duration of 1 day (OR: 48, 95% CI: 14–157, *P* < 0.001) was significantly associated with allergy. Specifically, urticaria fulfilling the ‘1-1-1’ criterion (appearance within 1 hour after the first dose and regression within 1 day) was reported by 122 of the 151 (80%) subjects found positive to allergy testing. Among the 77 patients unable to recall the two other relevant urticarial characteristics (i.e. dose and onset), a duration of at most 1 day was more frequent in those with positive tests (OR: 29, 95% CI: 6-141, *P* < 0.001). Among the 68 patients with a reaction during subsequent courses, 34 of the 42 (80%) patients who had had urticaria and 25 of the 26 (96%) who had experienced anaphylaxis reported 1-1-1 urticaria. Therefore, subjects with this type of urticaria have a high probability of experiencing a reaction more severe than the index reaction in case of re-exposure to the culprit penicillin and should be classified as being at high risk. There was no significant difference between subjects with positive and those with negative allergy testing regarding the time interval between the last β-lactam reaction and testing, with a median of 4 and 7 months, respectively. This finding suggests that in patients with an urticarial eruption, the duration of this time interval has limited relevance to risk stratification. However, the time intervals in this study were very low for both groups and do not reflect all populations. For example, most patients presenting for penicillin allergy assessment in the US where most evaluations are after 10 or more years.^48^ The results of a recent study concerning 1074 patients with immediate reactions to penicillins^49^ confirmed the importance of the duration of cutaneous reactions. Indeed, 123 (51.5%) of the 239 patients reporting urticaria, generalized erythema or local reactions to intramuscular injections with a duration of at most 1 day were positive to allergy tests. In contrast, only one (0.9%) of the 107 patients with cutaneous reactions lasting more than one day was positive. In the same study,^49^ the mean time interval between the most recent penicillin reaction and allergy examination was significantly longer (*P* < 0.0001) in the 444 patients with negative skin tests (mean 52.68 ± 98.96 months) than in the 630 patients with positive ones (mean 20.45 ± 68.13 months). Interestingly, 297 (99.7%) of the 298 patients who reported anaphylactic reactions that had occurred within 1 hour after the first dose, had regressed within 1 day and had been evaluated within 6 months tested positive in the allergy workup. This study confirmed that for reactions other than anaphylaxis, the time interval between index reactions and allergy testing has limited influence on the results.

After allergy history and risk assessment, drug allergy *in vivo* testing involves DCTs being considered the gold standard, preceded or not by skin testing. Severe reactions upon DCTs are exceedingly rare; in one meta-analysis of 112 studies including 26 595 participants, severe reactions occurred in just 0.06% and most severe reactions were anaphylaxis.^50^ The majority of allergies with well documented histories and records can be reliably ruled out and DCTs can be done with a full dose of the antibiotic. Before developing new diagnostic tools, *ex vivo* or *in vitro*, it is necessary to define the patients for whom a full challenge can be safely done since these patients may not need any form of pre-intervention diagnostic.

## Drug challenge tests

During a DCT a typical dose of a drug in the penicillin class is given with clinical observation ranging from 1 to 3 hours. If a single, full therapeutic dose is well-tolerated, there is negligible risk of a serious immediate reaction to a penicillin antibiotic, so penicillins can be used accordingly to known clinical application schemes in current and future treatment (use of other β-lactams such as cephalosporins, monobactams or carbapenems will depend on the patient’s other drug allergies). The DCT is considered as the current gold standard for excluding any IgE-mediated and delayed hyper-sensitivities towards penicillin, but a direct challenge (i.e. without previous skin testing) should be reserved for patients at low risk whereas a DCT should follow negative skin testing in moderate to high-risk phenotypes (Figure 3(a and b)). Mild-to-moderate delayed reactions, typically MPE, may still occur with a full penicillin course. Although more delayed cutaneous eruptions would be identified with a prolonged multi-day drug challenge, use of days of antibiotics without them being needed for treatment is not advised as this runs counter to antibiotic stewardship goals. Expansion of direct DCT programmes/procedures in low-risk patients should be considered as part of AMS programmes.^51^

## Penicillin skin testing (PST)

PST requires training; in most places, this specialized training is only given to allergists. However, nurses, nurse practitioners and non-allergist doctors can be trained to perform and interpret PST. In a meta-analysis of 27 studies, skin tests had a sensitivity of 30.7% and a specificity of 96.8%.^52^ The standard PST procedure is a multistep process using a panel of penicillin reagents: major (penicilloyl polylysine, also known as PPL or benzylpenicilloyl-octa-L-lysine), minor (penicillin G, penicilloate and penilloate), amoxicillin and possibly suspect β-lactams. A full test takes approximately 45–60 min to complete. There is regional variation among reagents available for PST. For example, in the US only PPL is commercially available since skin testing reagents are required to be FDA approved. Internationally, more reagents are available for standardized testing. Interpretation of skin tests remains subject to human expert observation that limits its use practically as allergy specialists are not uniformly available; in fact, in a study of 121 US hospitals from 38 US states, 44% had access to an allergist for inpatient consultations and 39% had access to inpatient PST.^53^ Both DCTs and PST are a favourable option that excludes IgE-mediated reactions with a more than 95% NPV.^54^ If such tests are negative, it is unlikely that a patient is allergic to penicillin.^36^ Conversely, desensitization should be considered if a drug is required in patients with proven or highly likely allergy and no alternative treatment is available as may be the case in severe infections. Desensitization, in its simplest format, consists in exposing the patient to a series of administrations in graded strengths of the substance to temporarily eliminate the hypersensitivity and thereby allowing the therapy despite allergy.

## Penicillin allergy diagnosis guidelines

Although penicillin allergy assessments are recognized as important, there is no globally agreed upon approach. Existing protocols evolve over time, differ among regions as they depend on penicillin and other β-lactam prescription patterns, change by organization of allergy services and vary in allergy testing reagents availability and usage. Notable efforts to converge are the joint collaboration of the American Academy of Allergy, Asthma & Immunology, the Infectious Disease Society of the Americas and the Society of Health Care Epidemiology of America, as well as the recent useful guidelines issued by the European Academy of Allergology and Clinical Immunology, both German and Austrian societies for Allergology and Clinical Immunology, the Canadian Society of Allergy and Clinical Immunology, the US Practical Guidance for the Evaluation and Management of Drug Hypersensitivity, and Australasian Society of Clinical Immunology and Allergy Consensus Statement For Assessment Of Immediate (IgE-Mediated) Penicillin Allergy.^6,7,55–57^ Still, these guidelines show substantial variation with respect to recommended reagents and diagnostic tools, protocols of the diagnostic methods (e.g. DCTs and skin tests), and diagnostic algorithms. For example, serum IgE assays and basophil activation tests (BATs) are not recommended in the US,^7,58^ whereas they are recommended as complementary tests in Europe.^6,55,59,60^

## Is there an unmet medical need?

The assessment of penicillin allergy remains a complex, and perhaps not entirely reliable process because it is often based on skin tests that involve manual steps and subjective interpretations. This assessment requires a multidisciplinary exercise as well as active participation of experts who are not routinely nor universally available and accessible. Capturing all patients with a penicillin allergy label is near impossible and would put a strain on healthcare setting operations and resources. When developing a PST service, it is important to consider the importance of education and training required and to customize the protocol based on local demand and target populations. Given that 10%–15% of hospitalized patients are estimated to have a possible penicillin allergy and that one in two hospitalized patients will receive an antibiotic during their hospital stay, there is a potential for a large volume of inpatients being eligible for PST. One US hospital estimated that >65 penicillin skin tests would need to be performed weekly if all eligible patients were skin tested. Consequently, this healthcare system adopted inpatient β-lactam care pathways that did not overly rely on skin testing.^61,62^ However, safe removal of the penicillin allergy label has a clear medical value in the inpatient setting and by enabling the appropriate use of penicillins is a welcome addition to the armamentarium of AMS interventions.

Penicillin skin testing is also cost-saving: several studies have demonstrated the cost savings associated with PST. Rimawi *et al.*^63^ estimated that the use of skin testing to guide antibiotic therapy yielded annual savings of US $82 000 for a group of 126 patients with a history of penicillin allergy at a 900-bed hospital. This is because penicillins can still be used instead of more costly and recent alternatives (β-lactamase inhibitors, 4th generation cephalosporins, aztreonam, carbapenems). Notably, aztreonam, a monobactam commonly used in penicillin-allergic patients, can cost up to $360/day versus $40/day for ceftriaxone.^64^ It has been determined that cost savings from shorter hospitalization duration would provide a more than nine-to-one return on investment in penicillin allergy testing.^8^ Additional evidence from a recent systematic review identified that inpatient costs were on average $1145 less for patients without penicillin allergy.^65^ In a simulation study considering both outpatients and inpatients in the US and Europe, penicillin allergy assessment was projected to be saving $657 for inpatients and $2746 for outpatients.^66^ In Australia, an inpatient penicillin allergy programme proved cost-effective.^25,67^

## How can we fill this gap?

### Clinical decision support systems

The initial clinical diagnosis of allergy risk is a process that would benefit from clinical decision support systems, since these can be locally parameterized with targeted populations, incidence of specific antibiotic allergies, local practice, etc. A recent review article highlights these benefits and defines barriers for their implementation.^68^ Interestingly, it has been shown that patients using a pilot computerized guideline had a significant 2-fold increased odds of receiving a penicillin or cephalosporin antibiotic despite a reported allergy.^69^ Those subjected to systematic skin testing (i.e. *in vivo* testing), however, had an almost 6-fold higher chance of receiving a penicillin or cephalosporin antibiotic.

PEN-FAST can be integrated in a decision tool.^42,44^ It calculates a score from three clinical parameters: (i) an allergy event occurring five or fewer years ago (2 points); (ii) anaphylaxis/angioedema or SCARs (2 points) and (iii) treatment required for an allergy episode (1 point). The results indicate that a score of less than three associated with a high NPV that can be used by clinicians and AMS professionals to identify low-risk penicillin allergies at the point of care. Previously published prediction tools, however, have been inconsistent in their predictions; it is not easy to clearly differentiate the different cutaneous reactions, and it is not clear whether the clinical history as such can be used accurately without *in vivo* or *in vitro* diagnostics.^70^ However, implementation of PEN-FAST or another history-driven prediction tool could be used to identify those patients with a risk low enough for a direct DCT. It is still advisable for AMS stakeholders to consider input or a close relationship with drug allergy specialists as some allergy histories will lie outside of any given criteria for risk stratification.

### In vitro diagnostic (IVD) testing

Immunochemical techniques can be used to detect sIgE (Type I immediate hypersensitivity) using formats involving coated antibiotic antigens. Examples of popular immunoassays are the radio-allergo-sorbent assay, ELISA or fluorescent immunoassay. Nowadays, use of IVD tests in penicillin allergy are less reliable compared to the *in vivo* penicillin allergy tests. The low sensitivity (38%–85%) and the lack of specificity of the currently available *in vitro* tests have several likely causes. First, the low concentration of antibiotic-sIgE in human serum is a primary issue. Second, there is variability to these tests in terms of quality and antigenic determinants used. All present immunoassays, including the commercial ones (i.e. the ‘Allergy’ tests from Siemens or ‘ImmunoCAP’ by Thermo-Fisher), are based exclusively on the detection of sIgE by means of the penicilloyl derivative determinants. Current manufacturers provide ‘specific’ tests for penicillin G, penicillin V, ampicillin and amoxicillin, all showing poor sensitivities and low numbers of true positives.^52,71–73^ Hence, IVD testing is at best supplementary to the *in vivo* tests. Also, false positive results with penicillin ImmunoCAP have been reported.^71,74^ In some cases, false positives were due to sIgE to a cross-reactive epitope, phenylethylamine, an allergenic structure related to penicillin.^51,64^ In other subjects, false positive results were explained by a nonspecific binding in the solid phase assay due to elevated total IgE titers.^75^ However, it was demonstrated that the use of a sIgE per total IgE ratio increased the ImmunoCAP specificity.^76^ In one study, among 171 subjects with histories of immediate allergic reactions to penicillins, 74 of the 80 subjects with values ≥0.002 of this ratio were allergic to penicillins, yielding a clinically useful positive predictive value of 92.5%. To further improve IVD testing performance, recent research has identified a new antigenic determinant for β-lactams, the ‘penamidyl’ epitope.^77^ An immunoassay has been developed, validated and applied successfully as a diagnostic tool for the detection of sIgE in the sera of 15 penicillin-allergic patients. This study demonstrates that developments are actively ongoing in the IVD field. Overall, sIgE assays to date have an overall summary sensitivity of 19.3% (95% CI, 12.0%–29.4%) and a specificity of 97.4% (95% CI, 95.2%–98.6%), with a partial area under the summary receiver-operating characteristic curve of 0.420 (*I*^2^ = 8.5%).^52^

### Ex vivo diagnostic testing

This type of laboratory analysis is mostly based on testing cells from the patient stimulated *in vitro* by penicillin exposure. Such tests are typically used in severe T-cell-mediated phenotypes.^78^ Type I immediate hypersensitivity can be assessed by the BAT or a histamine release test.^72^ Non-immediate hypersensitivity can be detected using lymphocyte transformation tests (LTT), IFN-y release enzyme-linked immunospot assay (ELISpot) or cellular allergen stimulation test (eCAST).^78^ In particular, the IFN-y release ELISpot can enhance the sensitivity of the diagnostic workup in patients reporting SCARs by assisting with the identification of the responsible antibiotics, including β-lactams^79^; however, its use remains primarily in research. These tests are not well standardized and their use is exclusively restricted to individual laboratories that apply their own methodologies. Their overall clinical utility is not well defined and to date there are no such IVD tests with regulatory approval.

## Patient and clinical perspective of ams integration

The act of spreading penicillin allergy diagnostic testing in itself is not sufficient to deliver the promised medical value. It has to be understood, endorsed and promoted by the clinician and understood by the patient. Recent studies highlight that patient awareness of penicillin allergy testing is significantly associated with completion of testing and that patients’ beliefs impact compliance to and completion of testing.^80^

If education favours the implementation of penicillin allergy testing, apprehension remains a barrier when using certain de-labelling assessment protocols: patients directly de-labelled (i.e. purely based on medical assessment or medication reconciliation thus not subjected to testing) were more likely to retain an antibiotic allergy label.^24^ Although they understood that their reaction was not an allergy, 33% would continue to avoid penicillins versus this figure was 5% for patients treated with another protocol that included *in vivo* testing.^24^ For a minority of patients not interested in penicillin testing, the most frequently cited reason was fear of adverse effects of testing.

On the clinician’s side, this population reported uncertainty about referral criteria for penicillin allergy testing. Following testing and a negative result, several clinicians remained reluctant to prescribe penicillins.^81^ This appeared to reflect a lack of confidence in the test result and fear of subsequent reactions to penicillins. The findings suggest again lack of awareness and knowledge of penicillin allergy testing services.

Both clinicians and patients need to be educated and supported in the use such services. Both groups should be equipped with the skills to use penicillins appropriately following a negative allergy test result.

## Recommendations

As the most frequently reported drug allergy globally, penicillin allergy has been shown to have substantial negative impact on patient outcomes, healthcare systems and antimicrobial resistance. Removal of the penicillin allergy label has a clear medical value and is now recognized as an inclusive part of AMS programmes. Improving the initial risk assessment of penicillin allergy is essential to help identify which patients need testing and what type of testing should be done. Performant risk stratification of patients in terms of low-, moderate- and high-risk allergy using validated clinical algorithms can be achieved and supported by different guidelines. The development of clinical decision support systems can standardize the approach and should be encouraged. This requires local customization based on target populations characteristics, incidence of specific allergies and local antibiotic usage. Further down the process, *in vivo* diagnostics using drug challenge and/or skin testing can safely rule out at-risk patients, and their expansion should be considered, as there is sufficient evidence that the cost of expert resources (allergist, trained pharmacist or infectious disease physician) are outweighed by the cost of benefits for patients and health systems. Still, given the complexity of *in vivo* diagnostics and widespread generalist lack of comfort with allergy, *in vitro* and *ex vivo* testing should be further investigated despite their current limitations in performance. Improved characterization of β-lactam antigenic determinants and other allergy biomarkers should be encouraged since these tests could offer a definitive operational advantage over *in vivo* testing and if implemented at scale could fulfil a large unmet medical need. By doing so and considering additional efforts in terms of developing awareness and providing better education for patients and clinicians, this will significantly contribute to an enhanced AMS impact.

## Transparency declarations

C. Mabilat, M.F. Gros, T.T. Timbrook and A. van Belkum are or were employees of bioMérieux at the time of writing. bioMérieux is a French multinational company developing and marketing *in vitro* diagnostic tests in the domain of infectious diseases. The opinions expressed in the current manuscript are those of the authors, which do not necessarily coincide with company policy. A. van Belkum is consulting for bioMérieux and currently works at BaseClear, a Dutch company in the next generation sequencing domain. A. Romano is consultant at Diater (Leganés, Madrid, Spain). All other authors: none to declare.
